# Deletion of *Bmal1* Prevents Diet-Induced Ectopic Fat Accumulation by Controlling Oxidative Capacity in the Skeletal Muscle

**DOI:** 10.3390/ijms19092813

**Published:** 2018-09-18

**Authors:** Taira Wada, Yuya Ichihashi, Emi Suzuki, Yasuhiro Kosuge, Kumiko Ishige, Taketo Uchiyama, Makoto Makishima, Reiko Nakao, Katsutaka Oishi, Shigeki Shimba

**Affiliations:** 1 Laboratory of Health Science, School of Pharmacy, Nihon University, 7-7-1 Narashinodai, Chiba, Funabshi 274-8555, Japan; wada.taira@nihon-u.ac.jp (T.W.); ichihashi.yuya.nusp@gmail.com (Y.I.); suzuki.emi.nusp@gmail.com (E.S.); 2 Laboratory of Pharmacology, School of Pharmacy, Nihon University, 7-7-1 Narashinodai, Chiba, Funabshi 274-8555, Japan; kosuge.yasuhiro@nihon-u.ac.jp (Y.K.); ishige.kumiko@nihon-u.ac.jp (K.I.); 3 Laboratory of Organic Chemistry, School of Pharmacy, Nihon University, 7-7-1 Narashinodai, Chiba, Funabshi 274-8555, Japan; uchiyama.taketo@nihon-u.ac.jp; 4 Division of Biochemistry, Department of Biomedical Sciences, School of Medicine, Nihon University, 30-1 Oyaguchi-Kamicho, Itabashi-ku, Tokyo 173-8610, Japan; makishima.makoto@nihon-u.ac.jp; 5 Biological Clock Research Group, Biomedical Research Institute, National Institute of Advanced Industrial Science and Technology (AIST), Tsukuba, Ibaraki 305-8566, Japan; nakao-reiko@tokushima-u.ac.jp (R.N.); k-ooishi@aist.go.jp (K.O.)

**Keywords:** skeletal muscle, circadian rhythm, ectopic fat, BMAL1

## Abstract

Brain and muscle arnt-like protein 1 (BMAL1), is a transcription factor known to regulate circadian rhythm. BMAL1 was originally characterized by its high expression in the skeletal muscle. Since the skeletal muscle is the dominant organ system in energy metabolism, the possible functions of BMAL1 in the skeletal muscle include the control of metabolism. Here, we established that its involvement in the regulation of oxidative capacity in the skeletal muscle. Muscle-specific *Bmal1* KO mice (MKO mice) displayed several physiological hallmarks for the increase of oxidative capacity. This included increased energy expenditure and oxygen consumption, high running endurance and resistance to obesity with improved metabolic profiles. Also, the phosphorylation status of AMP-activated protein kinase and its downstream signaling substrate acetyl-CoA carboxylase in the MKO mice were substantially higher than those in the *Bmal1*^flox/flox^ mice. In addition, biochemical and histological studies confirmed the substantial activation of oxidative fibers in the skeletal muscle of the MKO mice. The mechanism includes the regulation of *Cacna1s* expression, followed by the activation of calcium—nuclear factor of activated T cells (NFAT) axis. We thus conclude that BMAL1 is a critical regulator of the muscular fatty acid level under nutrition overloading and that the mechanism involves the control of oxidative capacity.

## 1. Introduction

Brain and muscle arnt-like protein 1 (BMAL1), also referred to as *Arntl*, *Arnt3* and, *MOP3* is a transcription factor that has a basic-helix-loop-helix (bHLH)/Per-Arnt-Sim (PAS) domain and regulates circadian rhythm of a spectrum of gene expressions [1,2,3]. BMAL1 forms heterodimers with another bHLH/PAS protein, circadian locomotor output cycles kaput (CLOCK) and the complex drives transcription from E-box elements found in the promoter of circadian-responsive genes including *period* (*Per*) and *cryptochrome* (*Cry*) [4,5,6].

Global *Bmal1*^−/−^ mice show several severe phenotypes, including changes in the rhythmicity of behavior, life span, body size, activity level and metabolic activity [7,8,9,10]. An extensive circadian cistrome analysis of the liver uncovered the presence of approx. 5900 BMAL1 binding sites in the genome [11,12,13,14]. The binding sites are associated with carbohydrate and lipid metabolism, transcriptional regulation and the cell cycle [11]. These results indicate that, in addition to the control of circadian rhythm in behavior, BMAL1 functions in the regulation of complex physiologic properties.

BMAL1 was originally characterized by its high expression in the brain and the skeletal muscle, suggesting the crucial roles in these tissues [1]. Indeed, several studies revealed the potential roles of BMAL1 in the regulation of muscular functions. First, the introduction of the *Bmal1* gene into the skeletal muscle of global *Bmal1* KO mice improved their activity level and body size of the mice [15]. Second, expression of the molecular circadian clock genes such as *Bmal1* and *Clock* and the genes related to muscle-specific functions showed a circadian rhythm in the skeletal muscle [16,17,18]. Importantly, most of the diurnal changes in these gene expressions were diminished in the *Clock* mutant mice [17]. Third, BMAL1 controls intracellular glucose metabolism through the regulation of pyruvate dehydrogenase activity [19,20]. Moreover, the deletion of *Bmal1* alone from adult skeletal muscle resulted in reductions in specific tension and increased muscle fibrosis [21].

Given the fact that the skeletal muscle is the dominant organ responsible for energy metabolism, a contribution of BMAL1 to the regulation of fatty acid metabolism is suggested. In this study, to better understand the role of BMAL1 in fatty acid metabolism in the skeletal muscle, mice with the specific deletion of *Bmal1* in the skeletal muscle (MKO mice) were subjected to a high fat diet (HFD) challenge. The results demonstrated that the deletion of *Bmal1* gene in the skeletal muscle prevents deposition of lipid and insulin resistance in obesity. The mechanism involves the increase of oxidative capacity and fatty acid oxidation activity.

## 2. Results

### 2.1. Deletion of Bmal1 Gene Has No Effects on the Body Weight, Behavior, or Muscular Structure

**Figure 1:**
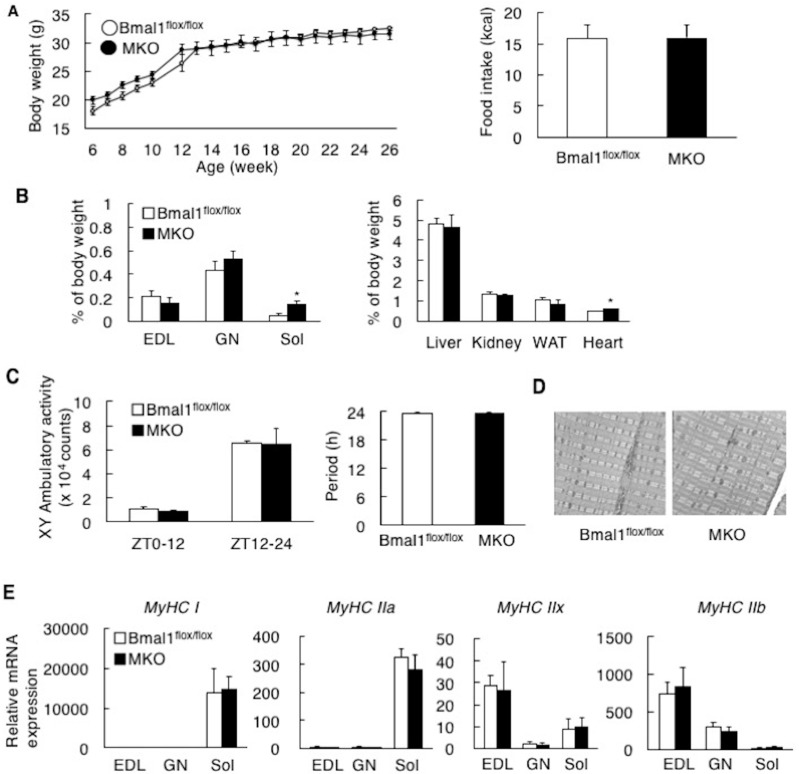
Deletion of *Bmal1* gene in the muscle has no effects on the body weight, behavior and muscular structure of the mice. (**A**) The body weights (left) and the daily calorie intake (right) of the *Bmal1*^flox/flox^ mice and muscle-specific *Bmal1* knockout (MKO) mice (*n* = 5). (**B**) The relative tissue weight to the body weight (*n* = 5). EDL, extensor digitorum longus. GN, gastrocnemius; Sol, soleus; WAT, white adipose tissue. (**C**) The free moving activity of male *Bmal1*^flox/flox^ mice and MKO mice measured with the infrared motion sensors (*n* = 11) (left). The period length of the *Bmal1*^flox/flox^ mice and MKO mice (*n* = 11) (right). (**D**) Electron micrographs of muscle cross-sections (× 4860). (**E**) Gene expressions level of myosin heavy chain isoforms in the *Bmal1*^flox/flox^ mice and MKO mice (*n* = 5). * *p* < 0.05 relative to *Bmal1*^flox/flox^ mice.

Muscle-specific *Bmal1* KO mice (MKO) were generated as described previously [10]. In all experiments, male *Bmal1*^flox/flox^ mice were used as the control. The phenotypes of the MKO mice showed both similarities and strikingly differences compared to those of the global *Bmal1* KO mice [8]. Regarding the body weight and food intake, there were no significant differences between the *Bmal1*^flox/flox^ mice and MKO mice (Figure 1A,B). However, our evaluation of the tissue weights revealed a significant increase in the weight of the soleus (Sol), as well as trends of increased gastrocnemius weight (GN) and decreased extensor digitorum longus (EDL) weight in the MKO mice compared to the *Bmal1*^flox/flox^ mice (Figure 1B). The tissue weight of the heart in the MKO mice was slightly but significantly heavier than that in the *Bmal1*^flox/flox^ mice (Figure 1B). Other tissue weight determined showed no significant differences (Figure 1B). The daily free-moving activity and the length of period in the MKO mice were comparable to those in the *Bmal1*^flox/flox^ mice (Figure 1C). This result is consistent with previous studies and confirms that the deletion of *Bmal1* in the skeletal muscle has no substantial effects on circadian pattern of behavior [21]. The structural analysis by electron microscopy and gene expression analysis of myosin heavy chain isoforms showed no significant differences between the two genotypes of mice (Figure 1D,E).

### 2.2. Deletion of Bmal1 Gene in the Muscle Increases Muscular Oxidative Capacity

**Figure 2:**
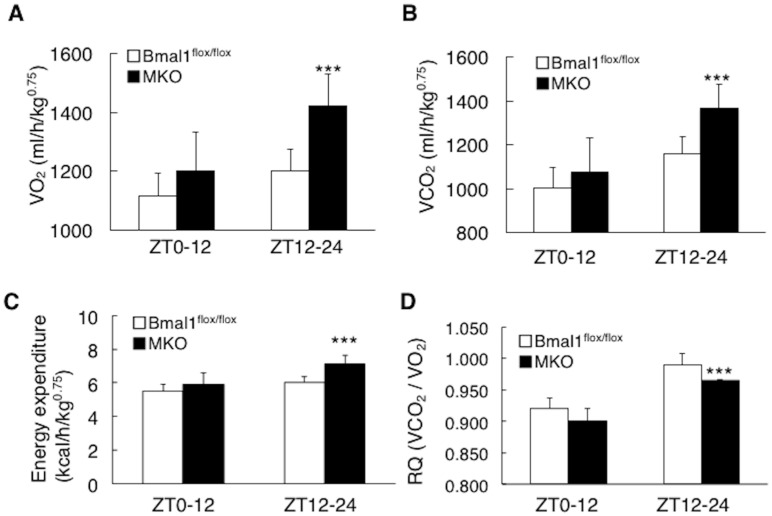
Deletion of the *Bmal1* gene in the muscle increased energy expenditure. (**A**) Oxygen consumption (VO_2_) (*n* = 5). (**B**) Carbon dioxide production (VCO_2_) (*n* = 5). (**C**) Energy expenditure. (**D**) Respiratory quotient (RQ). *** *p* < 0.001 relative to *Bmal1*^flox/flox^ mice.

**Figure 3:**
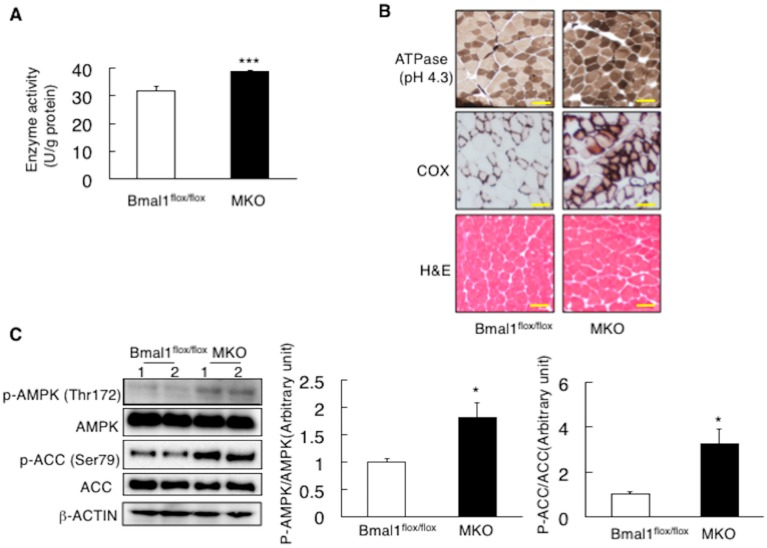

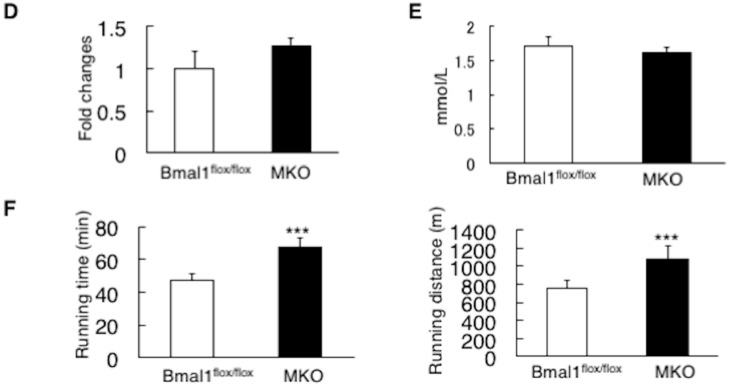
Deletion of *Bmal1* gene in the muscle increases muscular oxidative capacity. (**A**) Activity of 3-hydroxyacyl CoA dehydrogenase in the skeletal muscle at ZT10 (*n* = 6). (**B**) A representative image of the myosin-ATPase staining, the cytochrome c oxidase (COX) staining and hematoxylin & eosin (H&E) staining. Scale bar is 100 µm. (**C**) A representative Western blot of tissue extracts of the skeletal muscle at ZT10. Lanes 1 and 2 were run using samples from two different male mice (Left). Band intensity was analyzed with ImageJ (*n* = 4) (Right). (**D**) The mitochondrial DNA copy number in the skeletal muscle (*n* = 6). (**E**) Blood 3-hydroxybutyric acid level (*n* = 6). (**F**) Exercise training in the forced treadmill exercise test (*n* = 6). * *p* < 0.05, *** *p* < 0.001 relative to *Bmal1*^flox/flox^ mice.

To characterize the changes in energy metabolism resulting from the deficiency of *Bmal1* in the skeletal muscle, the factors associated with respiration were analyzed. As shown in Figure 2A,B, the value of O_2_ consumption and CO_2_ production in the MKO mice was higher than that in *Bmal1*^flox/flox^ mice during dark phase (Figure 2A,B). Also, during dark phase, the MKO mice displayed the higher energy expenditure and the lowered RQ value compared to the *Bmal1*^flox/flox^ mice (Figure 2C,D). The result in Figure 3A showed that the activities of 3-hydroxyacyl CoA dehydrogenase, an enzyme responsible for β-oxidation, in the MKO mice was higher than those in the *Bmal1*^flox/flox^ mice (Figure 3A). Histology with the staining of ATPase (pH 4.3) and cytochrome c oxidase confirmed the substantial activation of oxidative fibers in the MKO mice (Figure 3B). In the regulation of fatty acid oxidation, the activation of AMP-activated protein kinase (AMPK) is a key event. A western blot analysis revealed that the phosphorylation status of AMPK and its downstream signaling substrate acetyl-CoA carboxylase (ACC) in the MKO mice were substantially higher than those in the *Bmal1*^flox/flox^ mice (Figure 3C). With regards to the mitochondrial DNA copy number in the skeletal muscle, there was no significant difference between the *Bmal1*^flox/flox^ mice and MKO mice (Figure 3D). Also, the level of 3-hydroxybutyric acid in blood in MKO mice was almost equal to that in *Bmal1*^flox/flox^ mice (Figure 3E). In a last set of experiment, mice were run on treadmills until exhaustion. Strikingly, the running time and the distance of the MKO mice were able to sustain were significantly increased by 42% (Figure 3F).

### 2.3. Deletion of the Bmal1 Gene in the Muscle Improves the State of Diet-Induced Obesity

**Figure 4:**
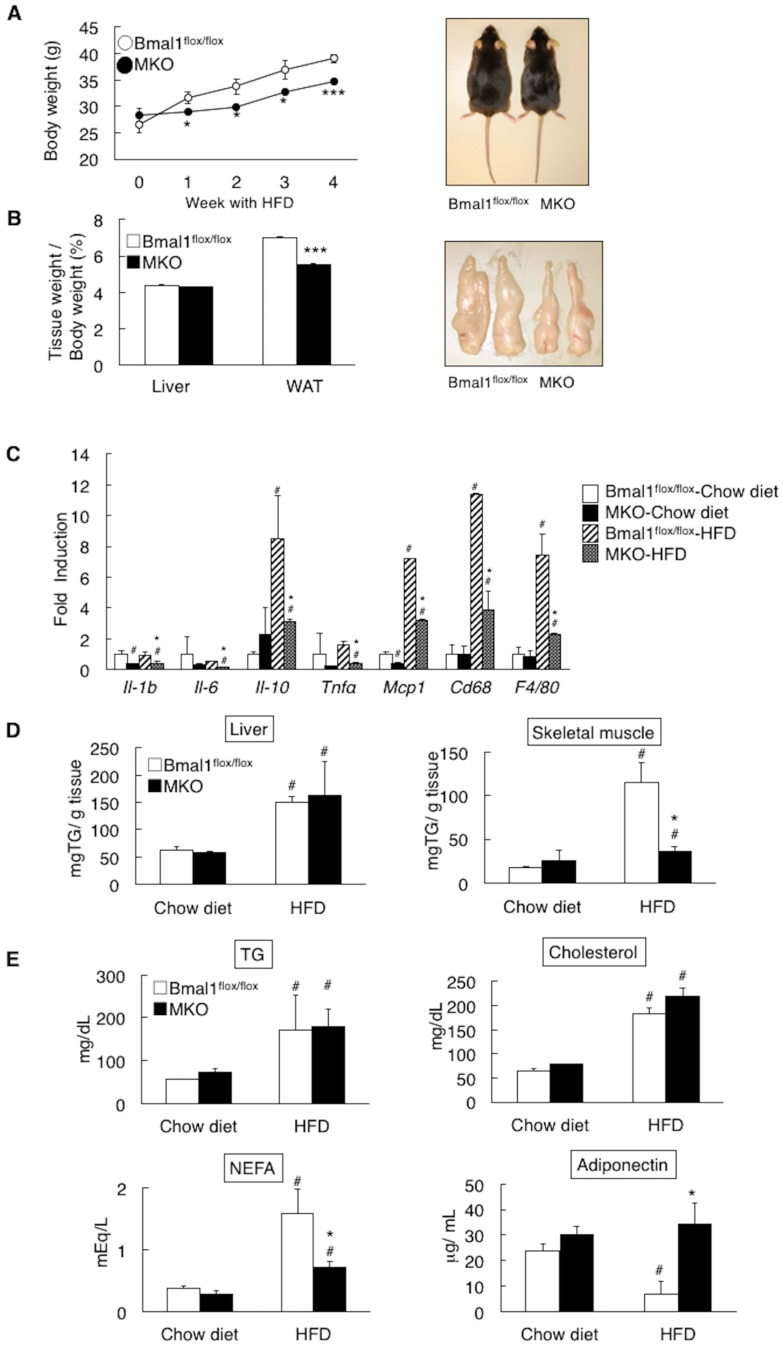
Deletion of the *Bmal1* gene in the muscle improves the state of obesity induced by high fat diet (HFD) feeding. (**A**) The body weight of male *Bmal1*^flox/flox^ mice and MKO mice under high fat diet (HFD) feeding condition (*n* = 6) (Left). A representative image of mice fed the HFD for 4 weeks (Right). (**B**) The relative tissue weight to body weight of the mice fed the HFD for 4 weeks (Left). A representative image of epididymal white adipose tissue of mice fed the HFD for 4 weeks (Right). (**C**) Gene expression in the white adipose tissue. # *p* < 0.05 relative to the *Bmal1*^flox/flox^ mice fed the chow diet. (**D**) Triglyceride (TG) contents in the liver (left) and the skeletal muscle (right) from male *Bmal1*^flox/flox^ mice and MKO mice fed the chow diet or HFD for 4 weeks. (**E**) Level of TG, cholesterol, non-esterified fatty acids (NEFA) and adiponectin in serum. In panel A and B, * *p* < 0.05, *** *p* < 0.001 relative to *Bmal1*^flox/flox^ mice. In panel (**C**–**E**), # *p* < 0.05 relative to the *Bmal1*^flox/flox^ mice fed the chow diet. * *p* < 0.05 relative to the *Bmal1*^flox/flox^ mice fed the HFD.

The high oxidative capacity may play a role in obesity resistance. Therefore, the obesity-related features of the MKO mice fed a high fat diet (HFD) were characterized. As shown in Figure 4A, the HFD feeding induced body weight gain in both the *Bmal1*^flox/flox^ mice and the MKO mice but the degree of weight gain in the MKO mice was smaller than that in the *Bmal1*^flox/flox^ mice (Figure 4A). The tissue weight of the white adipose tissue (WAT) in the MKO mice fed the HFD was less than that in the *Bmal1*^flox/flox^ mice (Figure 4B). Obesity induces inflammation in the adipose tissue [22]. The gene expression levels of inflammatory factors (*interleukins* and *Tnfa*) in the adipose tissue of the MKO mice were significantly lower than those in the *Bmal1*^flox/flox^ mice (Figure 4C). The HFD feeding increased the hepatic TG level in both the *Bmal1*^flox/flox^ mice and the MKO mice and the levels in the two mouse genotypes were comparable (Figure 4D). Ectopic fat accumulation was also observed in the skeletal muscle in both the *Bmal1*^flox/flox^ mice and MKO mice but the level in the MKO mice was far lesser than that in the *Bmal1*^flox/flox^ mice (Figure 4D). The levels of serum TG and cholesterol were elevated in the *Bmal1*^flox/flox^ mice and MKO mice by the HFD feeding and the levels showed no significant difference between the two genotypes (Figure 4E). The level of serum NEFA in the MKO mice was significantly lower than that in the *Bmal1*^flox/flox^ mice under the HFD feeding condition (Figure 4E). Obesity decreases the circulating adiponectin level, resulting in an increased risk of cardiovascular diseases [23,24]. The serum adiponectin level in the *Bmal1*^flox/flox^ mice was decreased by HFD feeding but the MKO mice showed steady adiponectin levels (Figure 4E).

### 2.4. Deletion of the Bmal1 Gene in the Muscle Improves the State of Insulin Sensitivity in Obesity

**Figure 5:**
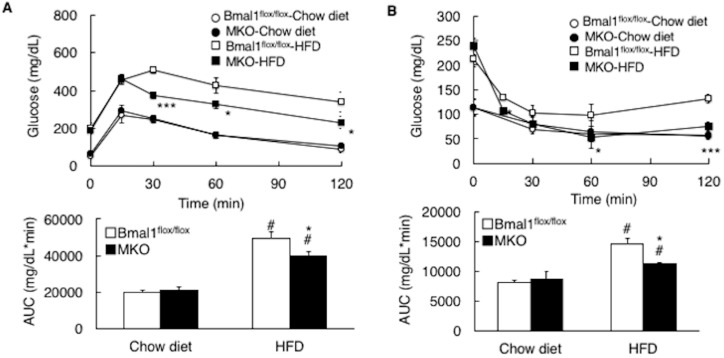

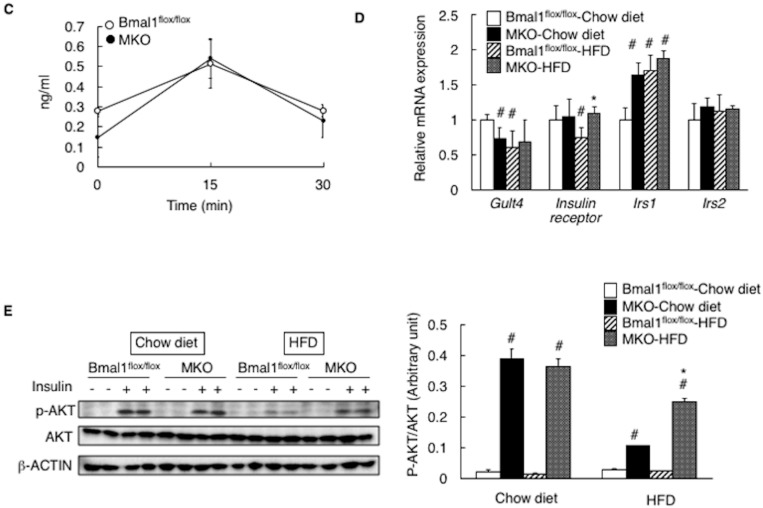
Deletion of the *Bmal1* gene in the muscle improves the state of insulin sensitivity in obesity. *Bmal1*^flox/flox^ mice and MKO mice were fed the chow diet or the HFD for 4 weeks before being analyzed (*n* = 6). (**A**) Glucose tolerance test. (**B**) Insulin tolerance test. The area under the curve (AUC) was calculated for respective group. (**C**) The level of serum insulin during the glucose tolerance test of mice fed the HFD for 4 weeks. (**D**) Gene expression in the skeletal muscle. (**E**) (Left) A representative Western blot of phosphorylated AKT (pAKT) and total AKT in the skeletal muscle of *Bmal1*^flox/flox^ mice and MKO mice. The skeletal muscle was isolated from mice after 15 min of insulin administration. (Right) Band intensity was analyzed with ImageJ (*n* = 4). In panel ((**A**,**B**) (*top*)) * *p* < 0.05, *** *p* < 0.001 relative to the *Bmal1*^flox/flox^ mice on the same diet. In panel ((**A**) (*bottom*), (**B**) (*bottom*)) and (**D**), # *p* < 0.05 relative to *Bmal1*^flox/flox^ mice fed the chow diet. * *p* < 0.05 relative to the *Bmal1*^flox/flox^ mice fed the HFD. In Panel (**E**), # *p* < 0.05 relative to the *Bmal1*^flox/flox^ mice fed the chow diet. * *p* < 0.05 relative to the *Bmal1*^flox/flox^ mice fed the HFD with insulin administration.

We next compared the whole-body glucose disposal rate between the two mouse genotypes. Under the condition of chow diet feeding, the score in the glucose tolerance test and insulin tolerance test in the MKO mice was comparable to that in the *Bmal1*^flox/flox^ mice (Figure 5A,B). The HFD feeding resulted in worse scores on these two parameters in both the *Bmal1*^flox/flox^ mice and MKO mice but the scores in the MKO mice were significantly better than those in the *Bmal1*^flox/flox^ mice (Figure 5A,B), while the insulin level during the glucose tolerance test in the MKO mice was almost equal to that in the *Bmal1*^flox/flox^ mice (Figure 5C). Previous studies reported the decrease of *glucose transporter 4* (*Glut4*) expression by the deletion of *Bmal1* in the skeletal muscle of mouse [19,20]. We also observed this reduction of *Glut4* expressions in the MKO mice fed the chow diet (Figure 5D). This study also observed the increase of *insulin receptor substrate 1* (*Irs1*) expression level in the MKO mice fed the chow diet (Figure 5D). Therefore, the effects of reduced expression of *Glut4* on insulin-dependent glucose uptake may be offset by increased expression of *Irs1*. Under the HFD condition, the change in the expression level of *Glut4* and *Irs1* was diminished (Figure 5D). The expression level of *insulin receptor* in the MKO mice was comparable to that in the *Bmal1*^flox/flox^ mice under chow diet condition. HFD feeding decreased the expression of *insulin receptor* in the *Bmal1*^flox/flox^ mice but not in the MKO mice (Figure 5D). To compare the intracellular insulin signal transduction activity between the *Bmal1*^flox/flox^ mice and MKO mice, the level of the insulin-induced phosphorylation status of protein kinase B (AKT) in the skeletal muscle was determined (Figure 5E). Similar to the results of the insulin tolerance tests (Figure 5B), the level of phosphorylated AKT in the MKO mice fed the chow diet was comparable to that in the *Bmal1*^flox/flox^ mice fed the chow diet (Figure 5E). In contrast, under the HFD feeding condition, the phosphorylated AKT level in the MKO mice was significantly higher than that in the *Bmal1*^flox/flox^ mice (Figure 5E).

### 2.5. Deletion of the Bmal1 Gene in the Muscle Induces Gene Expression Related to Lipid Metabolism

**Figure 6:**
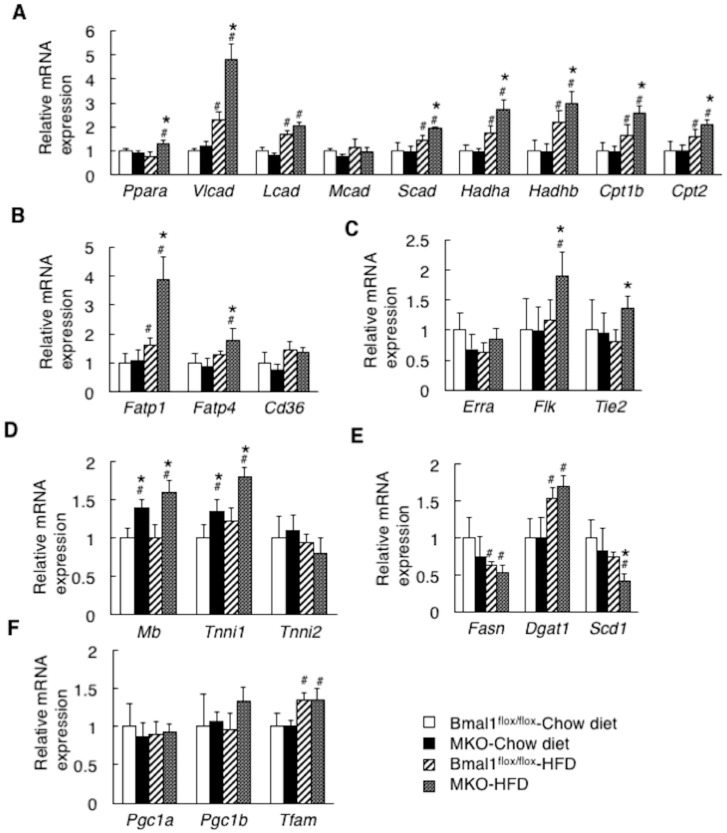
Deletion of *Bmal1* gene in the muscle increases the gene expression related to β-oxidation, fatty acid uptake and vascularization. *Bmal1*^flox/flox^ mice and MKO mice were fed the chow diet or the HFD for 4 weeks before being analyzed (*n* = 6). Gene expression in the mouse skeletal muscle was analyzed by qRT-PCR. (**A**) The expression of genes involved in β-oxidation. (**B**) The expression of genes involved in fatty acid uptake. (**C**) The expression of genes involved in vascularization. (**D**) The expression of myoglobin and troponin. (**E**) The expression of genes involved in lipogenesis. (**F**) The expression of genes involved in mitochondria biogenesis. # *p* < 0.05 relative to *Bmal1*^flox/flox^ mice fed the chow diet. * *p* < 0.05 relative to the *Bmal1*^flox/flox^ mice fed the HFD.

To understand the mechanism by which the deficiency of *Bmal1* improves the level of ectopic fat accumulation in the skeletal muscle (Figure 4D), we determined expression level of genes related to lipid metabolism by performing a qRT-PCR analysis (Figure 6). Under the HFD condition, the MKO mice showed an increased expression of β-oxidation-related genes, fatty acid uptake and vascularization. The genes include peroxisome *proliferator-activated receptor alpha* (*Ppara*), *very long-chain acyl-CoA dehydrogenase* (*Vlcad*), *short-chain acyl-CoAdehydrogenase* (*Scad*), *3-hydroxyacyl CoA dehydrogenase alpha and beta* (*Hadha and Hadhb*), *carnitine palmitoyltransferase 1 and 2* (*Cpt1 and 2*), *fatty acid transport protein 1 and 4* (*Fatp1 and 4*), *vascular endothelial growth factor receptor* (*Flk*) and *tyrosine kinase with Ig-like loops and Epidermal growth factor homology domains-2* (*Tie2*) (Figure 6A–C). The HFD feeding also increased the expression level of *myoglobin* (*Mb*) and *troponin I slow* (*Tnni1*) in the MKO mice (Figure 6D). In contrast, the gene expression of *stearoyl coenzyme decarboxylase1* (*Scd1*), a factor related to fatty acid synthesis, in the MKO mice was significantly lower than that in the *Bmal1*^flox/flox^ mice (Figure 6E). No significant difference was seen between the *Bmal1*^flox/flox^ mice and MKO mice in the expression level of genes responsible for mitochondria biosynthesis (Figure 6F).

### 2.6. Deletion of Bmal1 Gene Activates Calcium Signaling in the Skeletal Muscle

**Figure 7:**
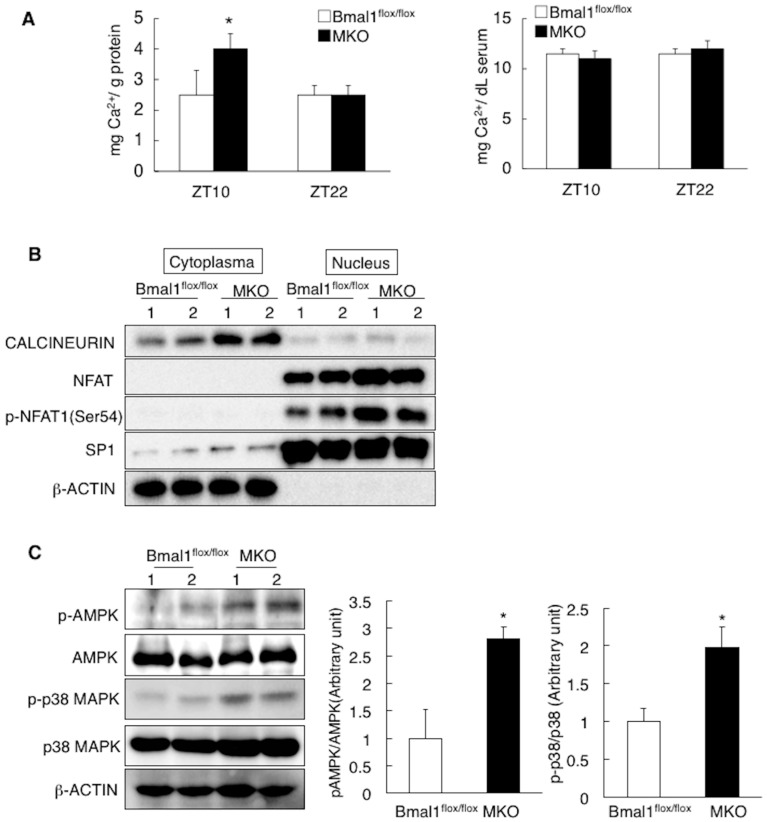
Deletion of *Bmal1* gene in the muscle activates calcium-dependent signaling pathway. (**A**) The level of Ca^2+^ in the skeletal muscles (left) and serum (right) at ZT10 and 22 (*n* = 6). (**B**) A representative Western blot of cytoplasmic and nuclear proteins of the skeletal muscle isolated at ZT10. (**C**) A representative Western blot of tissue extracts of the skeletal muscle isolated at ZT10 (Left). Band intensity was analyzed with ImageJ (*n* = 4) (Right). In panel (**B**,**C**), lanes 1 and 2 were run using samples from two different mice. * *p* < 0.05 relative to the *Bmal1*^flox/flox^ mice.

The results described above indicate that deletion of Bmal1 in the skeletal muscle increases oxidative capacity, resulting in suppression of diet-induced ectopic fat accumulation (Figure 3, Figure 4, Figure 5 and Figure 6). As shown in Figure 6D, gene expression level of slow fiber genes, such as myoglobin and troponin I slow, is elevated in MKO mice. Thus, to gain the insight by which deletion of Bmal1 increases oxidative capacity in the skeletal muscle, regulatory pathway involved in slow fiber gene expression was determined. Gene expression of slow fiber genes, such as myoglobin and troponin I slow, is regulated by calcium-nuclear factor of activated T cells (NFAT) axis [25,26]. In the MKO mice, the intra-muscular level of Ca^2+^ was greater than that in the *Bmal1*^flox/flox^ mice at ZT10, while no significant differences were observed in the serum Ca^2+^ level (Figure 7A). In response to the elevation of Ca^2+^ level, NFAT is translocated in the nucleus and phosphorylated at Ser 54 in the activation domain. This phosphorylation at Ser 54 increases the transcriptional activity of NFAT and thus the expression of the target genes such as myoglobin is induced [27]. Western blot analysis of the skeletal muscular proteins revealed accumulation of the phosphorylated-Ser54 form of NFAT1 in the nucleus in the MKO mice (Figure 7B). To increase slow fiber gene expression, phosphorylation of p38 mitogen-activated protein kinase (MAPK) via the activation of AMPK is also required [28,29]. The results in Figure 7C showed the increase in the phosphorylation status of AMPK and p38 MAPK.

**Figure 8:**
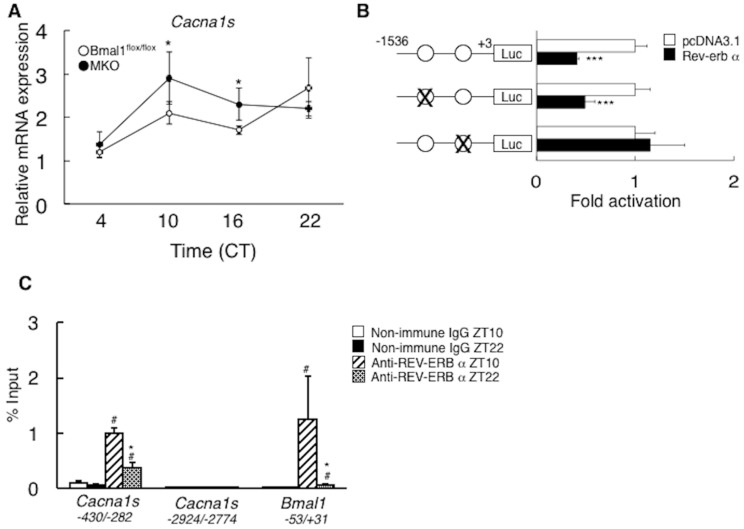
Cacna1s is a target gene of REV-REB α in the skeletal muscle. (**A**) Expressions of *Cacna1s* gene in the skeletal muscle of male *Bmal1*^flox/flox^ mice and MKO mice (*n* = 5). (**B**) Luciferase activity in HEK293 cells transfected with the reporter plasmids containing the *Cacna1s* promoter or its mutant variant in the presence of REV-ERB α or empty expression vector (pcDNA3.1) (*n* = 5). The positions of the putative RORE are labeled. Normalized luciferase activity in cells transfected with empty expression vector (pcDNA3.1) was arbitrarily set at 1. (**C**) ChIP analysis of the interaction between REV-ERB α and the region containing the *Cacna1s*/RORE in the mouse skeletal muscle at ZT10 and ZT22 (*n* = 4). * *p* < 0.05, *** *p* < 0.001 relative to the *Bmal1*^flox/flox^ mice (**A**), or pcDNA 3.1-transfected cells (**B**) (*t*-test). In panel (**C**), # *p* < 0.05 relative to non-immune IgG at ZT10. * *p* < 0.05 relative to anti-REV-ERB α antibody at ZT10.

In the MKO mice, the expression level of *Cacna1s*, a major calcium channel in the skeletal muscle, was significantly higher than that in the *Bmal1*^flox/flox^ mice (Figure 8A). The up-regulation of *Cacna1s* expression in the absence of *Bmal1* suggested that this gene expression might be under negative control of BMAL1, presumably via reverse orientation the c-erbA-1 gene (REV-ERB), a transcriptional suppressor. Reporter gene assay showed Cacna1s promoter activity is inhibited in the presence of REV-ERB α (Figure 8B). Inspection of this region identified two putative retinoic acid receptor-related orphan receptor-responsive element (RORE) at −1474/−1469 and −363/−358 upstream of the transcription start site. Introduction of a mutation in proximal RORE resulted in loss of responsiveness to the REV-ERB α (Figure 8B). The recruitment of the REV-ERB α to Cacna1s/RORE on the genome was confirmed by ChIP assay. In this ChIP assay, mouse Bmal1 promoter region containing RORE (−51 to −5) and a part of mouse Cacna1s gene (−2924 to −2774), which lacks RORE, were used as positive and negative controls, respectively. As shown in Figure 8C, the time-dependent recruitment of the REV-ERB α to the promoter region encompassing Cacna1s/RORE was observed in the skeletal muscle.

## 3. Discussion

BMAL1 was originally identified as a factor enriched in the skeletal muscle [1]. Rescue of *Bmal1* gene in the skeletal muscle of the global *Bmal1* KO mice improved the activity level [15], suggesting that BMAL1 plays the functional roles in the skeletal muscle. Since the skeletal muscle is the dominant organ system in energy metabolism, the possible functions of BMAL1 in the skeletal muscle include the control of metabolism. Moorsel et al. recently showed that fat oxidation in human skeletal muscle displays diurnal variation, with the highest activity at 8:00 a.m. and lowest activity at 11:00 p.m. [30]. This day-night shift of fat oxidation activity is inversely related to the level of *Bmal1* expression in human skeletal muscle [30]. The results in this study showed that mice lack *Bmal1* in the skeletal muscle display physiological hallmarks for the increase of oxidative capacity and fat oxidation activity. This included increased energy expenditure and oxygen consumption, high running endurance and resistance to obesity with improved metabolic profiles (Figure 2, Figure 3, Figure 4, Figure 5 and Figure 6). Also, biochemical and histological studies confirmed the substantial increase of oxidative activity in the skeletal muscle of the MKO mice (Figure 3A–C). Since no significant differences in daily free moving activity, the skeletal structure analyzed by electron microscopy and gene expression of myosin heavy chain isoforms were seen between the *Bmal1*^flox/flox^ and MKO mice (Figure 1), the marked increase in oxidative capacity represents the changes in metabolic activity in the skeletal muscle of the MKO mice. We were thus led to conclude that BMAL1 is a critical regulator of the muscular fatty acid level under nutrition overloading and that the mechanism involves the control of fatty acid oxidation.

Increase of oxidative capacity is at least partly accounted for by the elevated expression level of slow fiber genes, such as myoglobin and troponin I slow, in the MKO mice (Figure 6D). Expression of slow fiber genes is regulated by calcium-NFAT axis [26]. The results in Figure 7A showed the higher intra-muscular calcium level in MKO mice compared with *Bmal1*^flox/flox^ mice (Figure 7A). Also, activation of calcium-dependent signaling pathway was observed, as judged by phosphorylation status of NFAT1 (Ser54), AMPK and p38 MAPK, in the skeletal muscle of MKO mice (Figure 7B,C). Furthermore, these phenotypes of MKO mice are similar to those of transgenic mice overexpressing calcineurin, or calmodulin-dependent kinase [31,32]. Therefore, these results indicate that BMAL1 regulates calcium-NFAT axis in the skeletal muscle. Then we further analyzed the factors involved in calcium uptake in the skeletal muscle and found the increased expression of *Cacna1s* in MKO mice (Figure 8A). CACNA1S, a part of calcium channel, plays role in a signaling pathway determining muscle anabolic or catabolic state and might act as a molecular sensor of muscle activity [33]. Analysis of promoter region of *Cacna1s* revealed that REV-ERB α—a transcriptional suppressor—regulated by BMAL1, negatively regulates the expression of this gene (Figure 5B–D). *Rev-erb α* KO mice exhibit the increase of type I fiber in the skeletal muscle [34]. Consequently, we are led to conclude that BMAL1 regulates calcium signaling via the control of gene expression of *Cacna1S* by REV-ERB α and this activation of calcium-NFAT axis may be responsible for increase of the slow fiber genes expression and oxidative capacity in the MKO mice.

The regulation of metabolism by BMAL1 in peripheral tissues has been demonstrated by studies using a tissue-specific deletion of the *Bmal1* gene in mice. Hepatic BMAL1 drives a daily rhythm of hepatic glucose export timed so as to buffer the circulating glucose level [35]. The pancreatic clock regulates β-cell functions, including cell proliferation and insulin secretion [36]. In adipose tissue, deletion of the *Bmal1* gene results in obesity with a shift in the diurnal rhythm of food intake [37]. With regard to the skeletal muscle, metabolomics analysis revealed that deletion of the *Bmal1* gene leads to reduced glucose oxidation and a diversion of glycolytic intermediates to alternative metabolic pathway [19,20]. This could be partly responsible for the reduced ectopic fat accumulation in the skeletal muscle of MKO mice (Figure 2C). In addition, Schroder et al. showed that loss of *Bmal1* in the skeletal muscle increases oxidative fiber type [21]. In the present study, although no substantial increase in the oxidative fiber level was shown by the deletion of *Bmal1* in the skeletal muscle as judged by mRNA expression (Figure 1D), the MKO mice exhibited increased aerobic capacity (Figure 4 and Figure 5). These results indicate the activation of oxidative fibers in the skeletal muscle of MKO mice. Thus, BMAL1 is, at least partly, responsible for the regulation of muscle fiber activity and its related metabolic features.

The impaired cell structure and function of the skeletal muscle in the global *Bmal1* KO mice was demonstrated [9]. This includes disrupted myofilament and profound mitochondrial pathologies. We did not observe these abnormalities in the skeletal muscle of the MKO mice (Figure 1D). Muscular structure and functions depend not only on cellular status but also on neural innervations, motor neuron function and peripheral metabolic adaptation. As it has been reported, abnormal neuronal activities and immobilization are seen in the global *Bmal1* KO mice [7,10]. These past and present results suggest that tissue structure is partly controlled by a central clock via innervation. Alternatively, global *Bmal1* KO mice but not the MKO mice, exhibit the impaired insulin secretion [10,36]. The endocrinological differences might therefore generate the structural differences in the muscle between global *Bmal1* KO mice and MKO mice.

In this study, we compared the *Bmal1*^flox/flox^ mice and MKO mice at 16–18-week-old (Figure 1A). The results showed no substantial changes in body composition between genotypes under the chow diet condition. These results are also consistent with the reports by other groups. Dyer et al. also established the non-inducible and the inducible MKO mice [19]. In both lines of mice, no significant difference in body weight was observed by the deletion of *Bmal1*. The report by Esser’s group showed that the deletion of *Bmal1* had no effects on the body composition in 15–17 week-old mice, while the differences in body composition were observed in mice aged at 27 weeks or later [20]. Therefore, the effects of *Bmal1* deletion in the skeletal muscle varies with age. Further study is required to elucidate the age-dependent role of BMAL1 in the skeletal muscle.

An association between the molecular circadian clock system and muscular functions has long been suggested [16,17,18]. In the present study, we showed that BMAL1 regulates oxidative capacity, resulting in the control of the ectopic fat level and of insulin sensitivity in the skeletal muscle under over-nutrition conditions. Circadian misalignment in human such as shiftwork increases the risk of obesity and type 2 diabetes [38,39,40,41]. Our present findings showing the regulation of metabolic capacity by BMAL1 in the skeletal muscle provide additional insight into the link between obesity/diabetes and the role of the molecular circadian clock systems in energy metabolism.

## 4. Materials and Methods

### 4.1. Animals

All mice used in the experiments were male and 16–18 weeks old. Conditional *Bmal1*^flox/flox^ mice, which were generated using ES cells derived from C57BL/6J mice and MKO mice were generated as described [10]. In all experiments, littermate *Bmal1*^flox/flox^ mice were used as control mice. All mice were maintained at 23 ± 1 °C with 50 ± 10% relative humidity under a 12 h light/12 h dark cycle. Food and water were available ad libitum. The experimental protocol was approved by the Ethics Review Committee for Animal Experimentation of Nihon University (approval nos. AP11P009 (15/3/2011), AP12P018 (14/3/2012), AP13P032 (10/3/2013)). Animals were sacrificed and tissues were immediately excised to measure the tissue weight.

### 4.2. Locomotor Activity Rhythm Analysis

Wheel-running activity was continuously recorded using a Chronobiology Kit (Stanford Software Systems, Stanford, CA, USA). The free moving activity of the mice was recorded with the use of infrared motion sensors positioned directly above each cage and data were continuously recorded using an online system (Melquest, Ltd., Toyama, Japan).

### 4.3. Metabolic Studies

Energy expenditure was measured by using Oxyletpro system (PANLAB, S.L.U. Barcelona, Spain). The mice were subjected to glucose tolerance testing (GTT) and insulin tolerance tests (ITT) performed by administrating an intraperitoneal injection of dextrose solution (2 g/kg body weight for the mice fed the regular diet and 1 g/kg body weight for the mice fed the high fat diet) or insulin (0.5 U/kg body weight; Eli Lilly, Indianapolis, IN, USA), respectively. Mice were fasted 16 h in GTT and 6 h in ITT, respectively. Glucose levels were monitored before and after the injection with blood glucose strips (Arkray, Kyoto, Japan). Calcium were determined using a commercially available reagent.

### 4.4. Biochemical Analysis of Blood and Tissue

The blood levels of triglyceride, cholesterol, non-esterified fatty acid (Wako, Osaka, Japan), adiponectin (Otsuka Pharmaceutical Co., Ltd., Tokyo, Japan), 3-hydroxybutyric acid (Abbott Laboratories, IL, USA) and insulin, (Morinaga Institute of Biological Science, Inc., Kanagawa, Japan) were determined with commercial assay kit according to the manufacturer’s instructions.

### 4.5. Gene Expression (Quantitative Reverse Transcription Polymerase Chain Reaction [qRT-PCR])

Total RNA was extracted with RNAiso Plus (Takara Co., Ltd., Otsu, Japan) according to the manufacturer’s instructions. The cDNA was synthesized from 1.0 µg of total RNA by reverse transcriptase (Wako, Osaka, Japan). Aliquots of cDNA were amplified on a Stratagene MX3000 real-time PCR System using SYBR-Green PCR reagents (Promega, Madison, WI, USA). The mRNA expression levels were normalized against the *36B4* expression and are presented as relative expression levels. The primer sequences used are summarized in Table 1.

### 4.6. Exercise Performance Test

Prior to the exercise performance test, the mice were acclimated to the treadmill (Melquest, Ltd., Toyama, Japan) with a 5 min run at 7 m/min once per day for 2 days. The exercise test regimen was 10 m/min for the first 60 min, followed by incremental acceleration (1 m/min every 5 min) to a maximum speed of 20 m/min until exhaustion. Exhaustion was defined as failure to run for more than 20 s.

### 4.7. Histological Analyses

Skeletal muscle tissues were snap-frozen in a dry ice-acetone bath. Cryostat sections were stained with hematoxylin and eosin (H&E). Enzyme activity of myosin-ATPase and cytochrome c oxidase (COX) in frozen sections was evaluated as described [42,43].

### 4.8. Preparation of Tissue Extract

For the measurement of enzyme activity, the tissue was homogenized in the buffer (10 mM Hepes (pH 7.3), 11.5% sucrose, 0.1% TritonX-100 and 1 mM DTT) with a dounce grinder. After centrifugation for 10 min at 15,000× *g*, the resulting supernatant was subjected to an assay of the enzyme activity. For the whole tissue extract preparation, the tissue was homogenized in commercial lysis buffer (Cell Signaling Technology, Inc., Danvers, MA, USA) containing 1 mM DTT and phosphatase inhibitor (Roche Diagnostics K. K., Tokyo, Japan) with a dounce grinder. After centrifugation for 15 min at 15,000× *g*, the resulting supernatant was subjected to the experiments.

### 4.9. Measurement of 3-Hydroxyacyl CoA Dehydrogenase Activity

The maximal activity of 3-hydroxyacyl-CoA dehydrogenase in the muscle homogenate was assayed by monitoring the decrease in absorbance at 340 nm of NADH in the presence of acetoacetyl-CoA [44].

### 4.10. Electron Microscopy

The tissue samples were fixed with 2% paraformaldehyde and 2% glutaraldehyde in 0.1 M phosphate buffer (pH 7.4). The samples were then dehydrated, infiltrated and polymerized. Thin sections were cut and stained and images were obtained with a transmission electron microscope (JEM-1200EX; JEOL, Ltd., Tokyo, Japan) and a CCD camera (VELETA; Olympus Soft Imaging Solutions GmbH, Münster, Germany).

### 4.11. Western Blot Analysis

The proteins were resolved by sodium dodecyl sulfate-polyacrylamide gel electrophoresis (SDS-PAGE), transferred onto the membranes and probed with the antibodies. Immunoreactive proteins were visualized with ECL western blotting detection reagents (Thermo Fisher Scientific, Waltham, MA, USA). Antibodies against AKT and its phosphorylated form, AMPK and its phosphorylated form, Acetyl CoA carboxylase (ACC) and its phosphorylated form, CALCINURIN, NFAT and β-ACTIN were purchased from Cell Signaling Technology (Danvers, MA, USA). Antibodies against SP1 was purchased from Santa Cruz Biotechnology, Inc. (Dallas, TX, USA). Antibodies against phosphorylated (Ser54) NFAT were purchased from Sigma-Aldrich CO. (St. Louis, MO, USA).

### 4.12. Plasmid Construction, Cell Culture and Cell Transfection

HEK293 cells, obtained from Human Science Research Resources Bank (Osaka, Japan), were maintained in Dulbecco’s modified Eagle medium (DMEM) supplemented with 10% fetal bovine serum. The 5′ regulatory region (−1536 bp to +3 bp) of the mouse *Cacna1s* gene was amplified by PCR using mouse genomic DNA as the PCR template. The PCR-amplified fragment was cloned into the pGL3-basic vector (Promega). Site-directed mutagenesis was performed by the PCR overextension method and confirmed by DNA sequencing. HEK293 cells were transfected into 48-well plates with FuGene HD (Promega). After 16 h of incubation, the cells lysates were extracted and assayed with a dual luciferase reporter assay system (Promega). The pRL-tk vector (Promega), was used as a normalization control to correct for variable transfection efficiencies.

### 4.13. Chromatin Immunoprecipitation (ChIP) Assays

The chromatin immunoprecipitation (ChIP) assay was performed essentially as described elsewhere [45] with the modification for the skeletal muscle. In brief, the gastrocnemius of twelve-week-old C57BL/6J male mice was harvested at ZT10 and ZT22, cross-linked in 1% formaldehyde and lysed. The obtained tissue extracts were subjected to immunoprecipitation with an anti-REV-ERB α antibody (PPMX, Tokyo, Japan). Parallel samples were incubated with non-immune IgG as a negative control. The DNA region was amplified and quantitated by qPCR. The following PCR primers were used:

Cacna1s −430; 5′-AGAAACTTAATCTCCATCTAAGG-3′,
Cacna1s −282; 5′-GTGACTTATTATATCCAGGCTTG-3′,
Cacna1s −2924; 5′-TCCTCTGTAAAAAGCAGTACCTGC-3′,
Cacna1s −2774; 5′-CAGCCATTAATATCATTTCCTCTG-3′,
Bmal1 −53; 5′-GGAAAGTAGGTTAGTGGTGCGAC-3′,
and Bmal1 +31; 5′-AAGTCCGGCGCGGGTAAACAGG-3′.

### 4.14. Statistical Analysis

When applicable, the results are represented as the means ± SD. Statistical analysis was performed with Student’s *t*-test or a one-way analysis of variance (ANOVA) with Tukey’s post hoc test. A *p*-value of *p* < 0.05 was accepted as statistically significant.

## Figures and Tables

**Table 1:** Primer sequences.

Gene	Forward (5′-3′)	Reverse (5′-3′)
*36B4*	GCACTTTCGCTTTCTGGAGGGTGTC	TGACTTGGTGCTTTGGCGGGATTAG
*Cacna1s*	CCACACAGGGTAGCATGTAA	TCTCAGCTCCTCGTTAGCTT
*Cd36*	TGCTGGAGGTGTTATTGGTG	TGGGTTTTGCACATCAAAGA
*Cd68*	CTTCCCACAGGCAGCACAG	AATGATGAGAGGCAGCAAGAGG
*Cpt1b*	GTCGCTTCTTCAACGTCTGG	AAGAAAGCAGCACGTTCGAT
*Cpt2*	TCCTCGATCAAGATGGGAAC	GATCCTTCATCGGGAAGTCA
*Dgat1*	TGTGTGGTGATGCTGATCCTGAGT	GCCAGGCGCTTCTCAATCTGAAAT
*Erra*	GGAGGACGGCAGAAGTACAAA	GCGACACCAGAGCGTTCAC
*F4/80*	GTTTGGCTATGGGCTTCCAGTC	GCAAGGAGGACAGAGTTTATGGTG
*Fasn*	TGCTCCCAGCTGCAGGC	GCCCGGTAGCTCTGGGTGTA
*Fatp1*	TGGTCAAGGTCAATGAGGACACGA	ACGCTGTGGGCAATCTTCTTGTTG
*Fatp4*	TAGCCGCATCCTGTCCTTTGTGTA	CTTCTTGTTGTTGGCACCCTGGTT
*Flk*	CCCGCATGAAATTGAGCTAT	AAACATCTTCGCCACAGTCC
*Glut4*	GCTTTGTGGCCTTCTTTGAG	CGGCAAATAGAAGGAAGACG
*Hadha*	TGTGCCTGCTGCATTTGACA	ACAAGGCCTTTGCTCTGCTT
*Hadhb*	AGCTGCACTTTCGGGTTTGT	AACAGCTGTGGTCATGGCTT
*Il-1b*	CAACCAACAAGTGATATTCTCCATG	GATCCACACTCTCCAGCTGCA
*Il-6*	ACAACCACGGCCTTCCCTACT	CACGATTTCCCAGAGAACATG
*Il-10*	ACTTGGGTTGCCAAGCCTTA	AGAAATCGATGACAGCGCCT
*Insulin receptor*	AAAGTTTGCCCAACCATCTG	GTGAAGGTCTTGGCAGAAGC
*Irs1*	GCTCTAGTGCTTCCGTGTCC	GTTGCCACCCCTAGACAAAA
*Lcad*	ATGGCAAAATACTGGGCATC	TCTTGCGATCAGCTCTTTCA
*Mb*	CCTGGGTACCATCCTGAAGA	GAGCATCTGCTCCAAAGTCC
*Mcad*	CGCTCTTAGGACTACTTGCTAACC	ATGGTATTTACATGCAATGGACAG
*Mcp1*	CTTCTGGGCCTGCTGTTCA	CCAGCCTACTCATTGGGATCA
*MyHC I*	GCCTGGGCTTACCTCTCTATCAC	CTTCTCAGACTTCCGCAGGAA
*MyHC IIa*	CAGCTGCACCTTCTCGTTTG	CCCGAAAACGGCCATCT
*MyHC IIb*	CAATCAGGAACCTTCGGAACAC	GTCCTGGCCTCTGAGAGCAT
*MyHC IIx*	GGACCCACGGTCGAAGTTG	CCCGAAAACGGCCATCT
*Pgc1a*	GATGGCACGCAGCCCTAT	CTCGCACGGAGAGTTAAAGGAA
*Pgc1b*	AACCCAACCAGTCTCACACAGG	ATGCTGTCCTTGTGGGTAGG
*Ppara*	ATGCCAGTACTGCCCTTTTC	GGCCTTGACCTTGTTCATGT
*Scad*	CCACCAGACAAGACCGATTT	TCAATGAGGTATGGCACC
*Scd1*	TGGGTTGGCTGCTTGTG	GCGTGGGCAGGATGAAG
*Tfam*	CAAGTCAGCTGATGGGTATGG	TTTCCCTGAGCCGAATCATCC
*Tie2*	TTGAAGTGACGAATGAGAT	ATTTAGAGCTGTCTGGCTT
*Tnfa*	CGTCAGCCGATTTGCTATCT	CGGACTCCGCAAAGTCTAAG
*Tnni1*	TCATGCTGAAGAGCCTGATG	GGAGGCATTTGGCTTCAATA
*Tnni2*	CTGAGGGGCAAGTTCAATA	AGGTCCCGTTCCTTCTCAGT
*Vlcad*	TCATTGCCAAGGGCGGTTGAT	TTTGCTGATGGCGGCTTCTA

## Abbreviations

ACC	Acetyl-CoA carboxylase
AKT	Protein kinase B
AMPK	AMP-activated protein kinase.
BMAL	Brain and muscle arnt-like protein 1
Cd	Cluster of differentiation
Cpt	Carnitine palmitoyl transferase
Dgat1	Diacylglycerol O-acyltransferase 1
EDL	Extensor digitorum longus
Erra	Estrogen-related receptor-α
Fatp	Fatty acid transporter
Fasn	Fatty acid synthase
Flk	Fetal liver kinase
Glut4	Glucose transporter type 4
GN	Gastrocnemius
Hadh	Hydroxyacyl CoA dehydrogenase
HFD	High fat diet
Il	Interleukin
Irs	Insulin receptor substrate
Lcad	Long-chain acyl-CoA dehydrogenase
Mb	Myoglobin
Mcad	Medium-chain acyl-CoA dehydrogenase
Mcp1	Monocyte chemotactic protein-1
MyHC	Myosin heavy chain isoform
NFAT	Nuclear Factor of Activated T cells
Pgc-1	Peroxisome proliferator-activated receptor gammer coactivator-1
Ppar	Peroxisome proliferator-activated receptor
Rev-erb	Reverse orientation the c-erbA-1 gene
RORE	Retinoic acid receptor-related orphan receptor-responsive element
Scad	Short-chain acyl-CoA dehydrogenase
Scd1	Stearoyl-coA desaturase 1
Sol	Soleus
Tie2	TEK receptor tyrosine kinase 2
Tfam	Mitochondrial transcription factor A
Tnf	Tumor necrosis factor
Tnni1	Troponin I solow
Tnni2	Troponin I fast
Vlcad	Very long-chain acyl-CoA dehydrogenase
WAT	White adipose tissue
ZT	Zeitgeber time
